# *N*,*N*′-Dibenzyl-*N*′′-(2-chloro-2,2-difluoro­acet­yl)phospho­ric triamide

**DOI:** 10.1107/S1600536812039712

**Published:** 2012-09-26

**Authors:** Mehrdad Pourayoubi, Mojtaba Keikha, Jerry P. Jasinski, Amanda C. Keeley

**Affiliations:** a Department of Chemistry, Ferdowsi University of Mashhad, Mashhad, Iran; b Department of Chemistry, Keene State College, 229 Main Street, Keene, NH 03435-2001, USA

## Abstract

In the title mol­ecule, C_16_H_17_ClF_2_N_3_O_2_P, the N—H unit of the C(=O)NHP(=O) fragment adopts a *syn* orientation with respect to the P=O group. The two F atoms and the Cl atom of the ClF_2_C group are disordered over two sets of sites with refined occupancies of 0.605 (6) and 0.395 (6). In the crystal, mol­ecules are linked *via* N—H⋯O=C hydrogen bonds the and the (N—H⋯)(N—H⋯)O=P group into chains along [010].

## Related literature

For related structures with a P(=O)[NHC(=O)CClF_2_] fragment, and for reference values of P=O, C=O and P—N bond lengths and P—N—C bond angles, see: Pourayoubi *et al.* (2011 ▶); Raissi Shabari *et al.* (2011 ▶); Pourayoubi & Saneei (2011 ▶). For the double hydrogen-bond acceptor capability of the phosphoryl O atom in phospho­ramidates, see: Pourayoubi *et al.* (2012 ▶). For the synthesis of the starting material, CClF_2_C(=O)NHP(=O)Cl_2_, see: Iriarte *et al.* (2008 ▶).
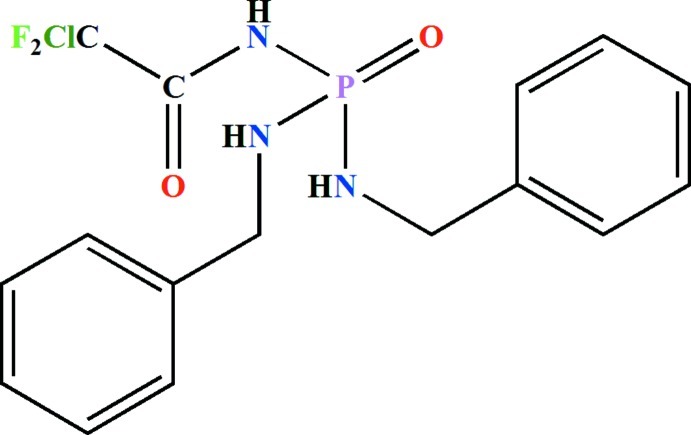


## Experimental

### 

#### Crystal data

C_16_H_17_ClF_2_N_3_O_2_P
*M* *_r_* = 387.75
Monoclinic, 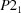

*a* = 12.9734 (5) Å
*b* = 4.9900 (2) Å
*c* = 13.7750 (4) Å
β = 96.482 (3)°
*V* = 886.06 (6) Å^3^
*Z* = 2
Mo *K*α radiation
μ = 0.34 mm^−1^
*T* = 173 K
0.35 × 0.22 × 0.12 mm

#### Data collection

Oxford Xcalibur (Eos, Gemini) diffractometer
Absorption correction: multi-scan (*CrysAlis RED*; Oxford Diffraction, 2010 ▶) *T*_min_ = 0.890, *T*_max_ = 0.960
9633 measured reflections
5460 independent reflections
4909 reflections with *I* > 2σ(*I*)
*R*_int_ = 0.025

#### Refinement

*R*[*F* ^2^ > 2σ(*F* ^2^)] = 0.051
*wR*(*F* ^2^) = 0.122
*S* = 1.09
5460 reflections
263 parameters
18 restraints
H atoms treated by a mixture of independent and constrained refinement
Δρ_max_ = 0.55 e Å^−3^
Δρ_min_ = −0.71 e Å^−3^
Absolute structure: Flack (1983 ▶), with 2216 Friedel pairs
Flack parameter: 0.06 (11)

### 

Data collection: *CrysAlis PRO* (Oxford Diffraction, 2010 ▶); cell refinement: *CrysAlis PRO*; data reduction: *CrysAlis RED* (Oxford Diffraction, 2010 ▶); program(s) used to solve structure: *SHELXS97* (Sheldrick, 2008 ▶); program(s) used to refine structure: *SHELXL97* (Sheldrick, 2008 ▶); molecular graphics: *SHELXTL* (Sheldrick, 2008 ▶); software used to prepare material for publication: *SHELXTL* and *enCIFer* (Allen *et al.*, 2004 ▶).

## Supplementary Material

Crystal structure: contains datablock(s) I, global. DOI: 10.1107/S1600536812039712/lh5506sup1.cif

Structure factors: contains datablock(s) I. DOI: 10.1107/S1600536812039712/lh5506Isup2.hkl

Additional supplementary materials:  crystallographic information; 3D view; checkCIF report

## Figures and Tables

**Table 1:** Hydrogen-bond geometry (Å, °)

*D*—H⋯*A*	*D*—H	H⋯*A*	*D*⋯*A*	*D*—H⋯*A*
N1—H1N⋯O1^i^	0.88 (2)	2.30 (3)	3.092 (3)	151 (3)
N2—H2N⋯O1^i^	0.86 (2)	2.05 (2)	2.867 (3)	158 (3)
N3—H3N⋯O2^ii^	0.86 (2)	2.01 (2)	2.854 (3)	166 (3)

Symmetry codes: (i) 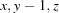; (ii) 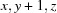.

**Fig. 1.**
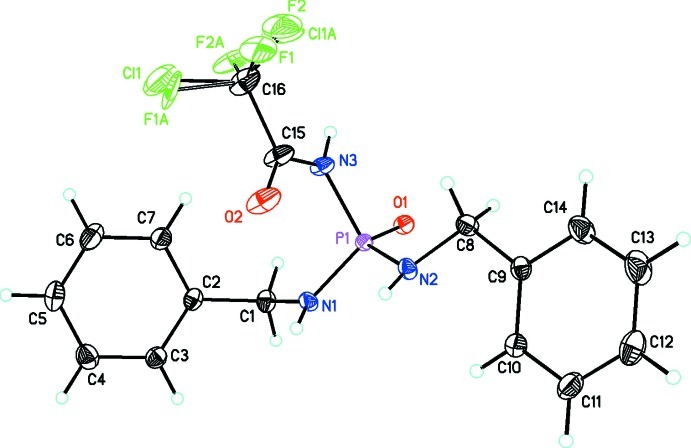
The molecular structure of the title compound. Displacement ellipsoids are given at the 30% probability level and H atoms are drawn as small spheres of arbitrary radii. The atoms of the minor component of disorder are labeled with suffix 'A'.

**Fig. 2.**
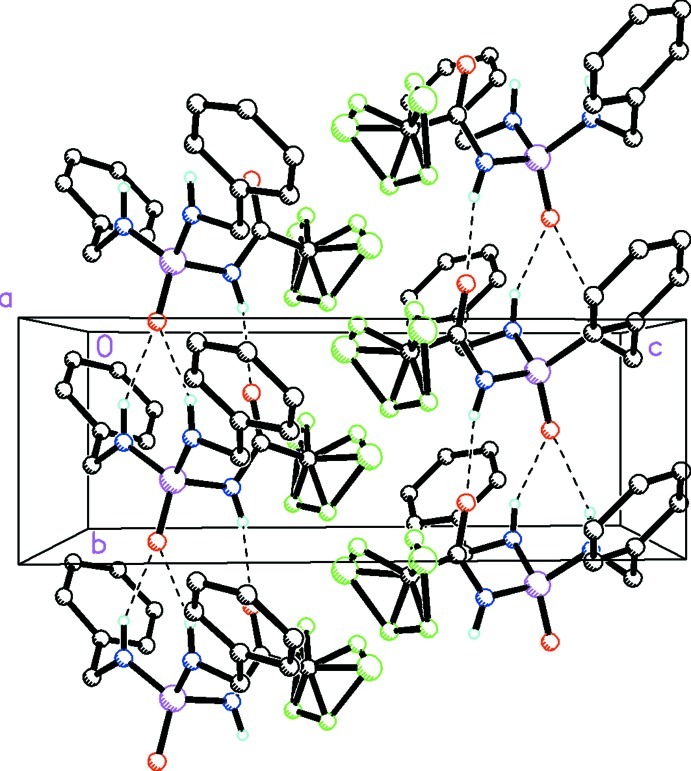
Crystal packing of title compound viewed approximately along the a axis. The N—H···O hydrogen bonds are shown by dashed lines. H atoms not involved in hydrogen bonding have been removed for clarity.

## supplementary crystallographic information

### Comment

In the previous studies, the structures of some compounds with a P(O)[NHC(O)CClF_2_] fragment have been investigated; for example, [4-CH_3_-C_6_H_4_NH]_2_P(O)[NHC(O)CClF_2_] (Pourayoubi, Tarahhomi *et al.*, 2011), [(C_6_H_5_CH_2_)(CH_3_)N]_2_P(O)[NHC(O)CClF_2_] (Raissi Shabari *et al.*, 2011) and [(CH_3_)_2_CHNH]_2_P(O)[NHC(O)CClF_2_] (Pourayoubi & Saneei, 2011). Here, the structure determination of the title compound (Fig. 1) is reported.

Atoms F1, F2 and Cl1 were refined as disordered over two sets of sites with occupancies of 0.605 (6) and 0.395 (6). The N—H unit of the C(O)NHP(O) fragment adopts a *syn* orientation with respect to the phosphoryl group. The P atom is bonded in a distorted tetrahedral environment as has been noted for other phosphoric triamides. The P═O, C═O and P—N bond lengths and P—N—C bond angles are within the expected values (Pourayoubi, Tarahhomi *et al.*, 2011; Raissi Shabari *et al.*, 2011; Pourayoubi & Saneei, 2011).

In the crystal, the O atom of P═O group acts as a double-hydrogen bond acceptor (Pourayoubi *et al.*, 2012) and molecules are linked by N—H···O═C hydrogen bonds and (N—H···)_2_O═P group, into a linear arrangement along the *b* axis (Fig. 2).

### Experimental

ClF_2_CC(O)NHP(O)Cl_2_ was prepared according to the literature method reported by Iriarte *et al.* (2008).

To a solution of ClF_2_CC(O)NHP(O)Cl_2_ (0.473 g, 1.92 mmol) in dry chloroform (25 ml), a solution of benzylamine (0.823 g, 7.68 mmol) in the same solvent (5 ml) was added at 273 K. After 6 h stirring, the solvent was removed and the product was washed with distilled water and recrystallized from CH_3_CN at room temperature.

### Refinement

H atoms H1N, H2N and H3N were located in a difference Fourier map and were refined with U_iso_(H) = 1.2U_eq_(N), giving N—H distances of 0.88 (2) or 0.86 (2) Å. The other H atoms were placed in calculated positions with 0.95 Å for CH, 0.99 Å for CH_2_ and with U_iso_(H) = 1.2U_eq_(C). F atoms F1 and F2 and chlorine Cl1 are disordered over two sets of sites with occupancies of 0.605 (6) and 0.395 (6).

### Crystal data

**Table:**

C_16_H_17_ClF_2_N_3_O_2_P	*F*(000) = 400
*M**_r_* = 387.75	*D*_x_ = 1.453 Mg m^−^^3^
Monoclinic, *P*2_1_	Mo *K*α radiation, λ = 0.71073 Å
Hall symbol: P 2yb	Cell parameters from 3176 reflections
*a* = 12.9734 (5) Å	θ = 3.3–32.2°
*b* = 4.9900 (2) Å	µ = 0.34 mm^−^^1^
*c* = 13.7750 (4) Å	*T* = 173 K
β = 96.482 (3)°	Block, colourless
*V* = 886.06 (6) Å^3^	0.35 × 0.22 × 0.12 mm
*Z* = 2	

### Data collection

**Table:**

Oxford Xcalibur (Eos, Gemini) diffractometer	5460 independent reflections
Radiation source: Enhance (Mo) X-ray Source	4909 reflections with *I* > 2σ(*I*)
Graphite monochromator	*R*_int_ = 0.025
Detector resolution: 16.1500 pixels mm^-1^	θ_max_ = 32.2°, θ_min_ = 3.3°
ω scans	*h* = −18→19
Absorption correction: multi-scan (*CrysAlis RED*; Oxford Diffraction, 2010)	*k* = −7→6
*T*_min_ = 0.890, *T*_max_ = 0.960	*l* = −9→20
9633 measured reflections	

### Refinement

**Table:**

Refinement on *F*^2^	Secondary atom site location: difference Fourier map
Least-squares matrix: full	Hydrogen site location: inferred from neighbouring sites
*R*[*F*^2^ > 2σ(*F*^2^)] = 0.051	H atoms treated by a mixture of independent and constrained refinement
*wR*(*F*^2^) = 0.122	*w* = 1/[σ^2^(*F*_o_^2^) + (0.048*P*)^2^ + 0.3968*P*] where *P* = (*F*_o_^2^ + 2*F*_c_^2^)/3
*S* = 1.09	(Δ/σ)_max_ = 0.001
5460 reflections	Δρ_max_ = 0.55 e Å^−^^3^
263 parameters	Δρ_min_ = −0.71 e Å^−^^3^
18 restraints	Absolute structure: Flack (1983), with 2216 Friedel pairs
Primary atom site location: structure-invariant direct methods	Flack parameter: 0.06 (11)

### Special details

**Table:**

Experimental. IR (KBr, ν, cm^-1^): 3253, 1718, 1457, 1419, 1282, 1215, 1139, 1073, 977, 873, 735 and 688.
Geometry. All e.s.d.'s (except the e.s.d. in the dihedral angle between two l.s. planes) are estimated using the full covariance matrix. The cell e.s.d.'s are taken into account individually in the estimation of e.s.d.'s in distances, angles and torsion angles; correlations between e.s.d.'s in cell parameters are only used when they are defined by crystal symmetry. An approximate (isotropic) treatment of cell e.s.d.'s is used for estimating e.s.d.'s involving l.s. planes.
Refinement. Refinement of *F*^2^ against ALL reflections. The weighted *R*-factor *wR* and goodness of fit *S* are based on *F*^2^, conventional *R*-factors *R* are based on *F*, with *F* set to zero for negative *F*^2^. The threshold expression of *F*^2^ > σ(*F*^2^) is used only for calculating *R*-factors(gt) *etc*. and is not relevant to the choice of reflections for refinement. *R*-factors based on *F*^2^ are statistically about twice as large as those based on *F*, and *R*-factors based on ALL data will be even larger.

### Fractional atomic coordinates and isotropic or equivalent isotropic displacement parameters (Å^2^)

**Table:**

	*x*	*y*	*z*	*U*_iso_*/*U*_eq_	Occ. (<1)
P1	0.51645 (5)	0.70594 (13)	0.19236 (4)	0.01939 (13)	
Cl1	0.2161 (2)	0.5360 (8)	0.3959 (2)	0.0795 (10)	0.605 (6)
F1	0.3784 (5)	0.5030 (15)	0.4995 (4)	0.086 (2)	0.605 (6)
F2	0.3555 (5)	0.8763 (7)	0.4479 (3)	0.073 (2)	0.605 (6)
Cl1A	0.3973 (4)	0.6102 (13)	0.5203 (3)	0.0829 (18)	0.395 (6)
F1A	0.2518 (6)	0.4471 (17)	0.4089 (7)	0.066 (3)	0.395 (6)
F2A	0.2775 (5)	0.8461 (12)	0.3861 (5)	0.058 (2)	0.395 (6)
O1	0.54403 (15)	0.9827 (4)	0.16821 (14)	0.0261 (4)	
O2	0.4173 (2)	0.3092 (5)	0.3189 (2)	0.0528 (8)	
N1	0.45241 (18)	0.5364 (5)	0.10546 (18)	0.0266 (5)	
H1N	0.460 (3)	0.362 (5)	0.106 (3)	0.032*	
N2	0.61194 (18)	0.5089 (5)	0.22963 (16)	0.0241 (4)	
H2N	0.601 (3)	0.340 (5)	0.226 (2)	0.029*	
N3	0.4401 (2)	0.7487 (5)	0.28612 (18)	0.0288 (5)	
H3N	0.428 (3)	0.909 (5)	0.305 (2)	0.035*	
C1	0.3635 (2)	0.6492 (6)	0.0436 (2)	0.0294 (6)	
H1B	0.3792	0.6516	−0.0251	0.035*	
H1A	0.3527	0.8367	0.0637	0.035*	
C2	0.2652 (2)	0.4925 (6)	0.04980 (19)	0.0270 (5)	
C3	0.2351 (2)	0.2941 (6)	−0.0179 (2)	0.0309 (6)	
H3A	0.2759	0.2598	−0.0696	0.037*	
C4	0.1463 (3)	0.1456 (7)	−0.0111 (3)	0.0391 (7)	
H4A	0.1267	0.0093	−0.0577	0.047*	
C5	0.0860 (2)	0.1951 (9)	0.0635 (2)	0.0432 (7)	
H5A	0.0247	0.0937	0.0679	0.052*	
C6	0.1150 (3)	0.3920 (9)	0.1315 (3)	0.0457 (9)	
H6A	0.0742	0.4238	0.1835	0.055*	
C7	0.2035 (2)	0.5440 (8)	0.1241 (2)	0.0364 (7)	
H7A	0.2219	0.6832	0.1698	0.044*	
C8	0.6884 (2)	0.5876 (6)	0.3112 (2)	0.0286 (6)	
H8A	0.6577	0.5637	0.3733	0.034*	
H8B	0.7050	0.7800	0.3048	0.034*	
C9	0.7871 (2)	0.4274 (6)	0.31541 (19)	0.0263 (5)	
C10	0.8070 (2)	0.2410 (7)	0.2450 (2)	0.0303 (6)	
H10A	0.7574	0.2120	0.1899	0.036*	
C11	0.8993 (3)	0.0966 (8)	0.2550 (3)	0.0401 (7)	
H11A	0.9121	−0.0311	0.2067	0.048*	
C12	0.9720 (3)	0.1367 (8)	0.3341 (3)	0.0453 (9)	
H12A	1.0345	0.0358	0.3409	0.054*	
C13	0.9537 (3)	0.3244 (9)	0.4035 (3)	0.0505 (10)	
H13A	1.0045	0.3561	0.4575	0.061*	
C14	0.8624 (3)	0.4658 (8)	0.3949 (2)	0.0403 (7)	
H14A	0.8502	0.5920	0.4439	0.048*	
C15	0.4032 (3)	0.5428 (6)	0.3344 (3)	0.0405 (8)	
C16	0.3359 (2)	0.6241 (6)	0.4143 (2)	0.0490 (8)	

### Atomic displacement parameters (Å^2^)

**Table:**

	*U*^11^	*U*^22^	*U*^33^	*U*^12^	*U*^13^	*U*^23^
P1	0.0211 (3)	0.0136 (2)	0.0242 (3)	−0.0009 (3)	0.00582 (19)	−0.0014 (2)
Cl1	0.0438 (12)	0.139 (3)	0.0597 (12)	0.0070 (14)	0.0255 (10)	−0.0081 (14)
F1	0.076 (4)	0.169 (7)	0.0121 (19)	−0.017 (4)	0.0062 (19)	−0.016 (3)
F2	0.139 (5)	0.036 (2)	0.056 (3)	−0.013 (3)	0.066 (3)	−0.0126 (18)
Cl1A	0.076 (3)	0.148 (5)	0.0257 (15)	0.005 (3)	0.0091 (13)	−0.0213 (17)
F1A	0.028 (3)	0.079 (6)	0.098 (6)	−0.017 (4)	0.036 (4)	0.007 (5)
F2A	0.081 (5)	0.043 (3)	0.060 (4)	0.032 (3)	0.047 (4)	0.012 (3)
O1	0.0319 (10)	0.0160 (9)	0.0322 (9)	−0.0018 (7)	0.0107 (7)	0.0004 (7)
O2	0.0767 (19)	0.0144 (10)	0.0769 (18)	0.0017 (12)	0.0504 (16)	0.0025 (11)
N1	0.0229 (10)	0.0199 (11)	0.0361 (11)	0.0025 (9)	−0.0006 (8)	−0.0079 (9)
N2	0.0240 (10)	0.0158 (10)	0.0316 (11)	−0.0011 (8)	−0.0008 (8)	−0.0015 (9)
N3	0.0405 (13)	0.0129 (12)	0.0366 (11)	0.0009 (9)	0.0198 (10)	−0.0012 (8)
C1	0.0263 (12)	0.0335 (18)	0.0281 (12)	−0.0024 (10)	0.0009 (9)	0.0050 (10)
C2	0.0218 (12)	0.0308 (14)	0.0281 (12)	0.0024 (10)	0.0014 (9)	0.0067 (10)
C3	0.0275 (13)	0.0332 (16)	0.0321 (13)	0.0026 (11)	0.0033 (11)	0.0008 (11)
C4	0.0366 (16)	0.0320 (19)	0.0477 (17)	−0.0011 (12)	0.0000 (13)	−0.0013 (12)
C5	0.0271 (13)	0.0458 (19)	0.0568 (18)	−0.0050 (17)	0.0047 (12)	0.008 (2)
C6	0.0327 (17)	0.064 (3)	0.0427 (17)	−0.0004 (17)	0.0129 (13)	0.0049 (17)
C7	0.0287 (14)	0.049 (2)	0.0314 (13)	−0.0020 (14)	0.0042 (11)	−0.0040 (13)
C8	0.0296 (13)	0.0269 (14)	0.0284 (12)	0.0019 (11)	−0.0006 (10)	−0.0036 (10)
C9	0.0252 (12)	0.0265 (13)	0.0270 (12)	−0.0032 (10)	0.0025 (9)	0.0035 (10)
C10	0.0260 (12)	0.0340 (17)	0.0309 (12)	−0.0004 (12)	0.0030 (9)	−0.0017 (11)
C11	0.0298 (15)	0.0427 (19)	0.0494 (18)	0.0062 (14)	0.0113 (13)	−0.0021 (15)
C12	0.0297 (15)	0.052 (2)	0.0533 (19)	0.0088 (14)	0.0011 (13)	0.0121 (16)
C13	0.0378 (19)	0.062 (3)	0.0474 (19)	0.0040 (17)	−0.0156 (15)	0.0013 (18)
C14	0.0393 (17)	0.0429 (19)	0.0356 (15)	0.0020 (15)	−0.0096 (12)	−0.0027 (14)
C15	0.060 (2)	0.0165 (13)	0.0516 (18)	0.0013 (13)	0.0366 (16)	0.0015 (12)
C16	0.0641 (16)	0.0245 (15)	0.066 (2)	−0.0012 (15)	0.0394 (15)	0.0048 (15)

### Geometric parameters (Å, º)

**Table:**

P1—O1	1.474 (2)	C3—H3A	0.9500
P1—N1	1.617 (2)	C4—C5	1.382 (5)
P1—N2	1.619 (2)	C4—H4A	0.9500
P1—N3	1.728 (2)	C5—C6	1.381 (6)
Cl1—C16	1.607 (3)	C5—H5A	0.9500
F1—C16	1.379 (5)	C6—C7	1.390 (5)
F2—C16	1.355 (4)	C6—H6A	0.9500
Cl1A—C16	1.585 (4)	C7—H7A	0.9500
F1A—C16	1.399 (5)	C8—C9	1.505 (4)
F2A—C16	1.373 (4)	C8—H8A	0.9900
O2—C15	1.203 (4)	C8—H8B	0.9900
N1—C1	1.467 (4)	C9—C10	1.388 (4)
N1—H1N	0.88 (2)	C9—C14	1.396 (4)
N2—C8	1.466 (3)	C10—C11	1.391 (4)
N2—H2N	0.86 (2)	C10—H10A	0.9500
N3—C15	1.341 (4)	C11—C12	1.373 (5)
N3—H3N	0.86 (2)	C11—H11A	0.9500
C1—C2	1.507 (4)	C12—C13	1.378 (6)
C1—H1B	0.9900	C12—H12A	0.9500
C1—H1A	0.9900	C13—C14	1.373 (5)
C2—C3	1.386 (4)	C13—H13A	0.9500
C2—C7	1.393 (4)	C14—H14A	0.9500
C3—C4	1.382 (4)	C15—C16	1.534 (4)
			
O1—P1—N1	116.13 (13)	C9—C8—H8B	109.0
O1—P1—N2	116.36 (12)	H8A—C8—H8B	107.8
N1—P1—N2	103.17 (12)	C10—C9—C14	118.1 (3)
O1—P1—N3	103.15 (11)	C10—C9—C8	123.6 (2)
N1—P1—N3	109.14 (13)	C14—C9—C8	118.3 (3)
N2—P1—N3	108.72 (12)	C9—C10—C11	120.2 (3)
C1—N1—P1	122.3 (2)	C9—C10—H10A	119.9
C1—N1—H1N	118 (2)	C11—C10—H10A	119.9
P1—N1—H1N	117 (2)	C12—C11—C10	120.7 (3)
C8—N2—P1	120.50 (19)	C12—C11—H11A	119.7
C8—N2—H2N	114 (2)	C10—C11—H11A	119.7
P1—N2—H2N	118 (2)	C11—C12—C13	119.5 (3)
C15—N3—P1	122.91 (19)	C11—C12—H12A	120.2
C15—N3—H3N	119 (2)	C13—C12—H12A	120.2
P1—N3—H3N	118 (2)	C14—C13—C12	120.3 (3)
N1—C1—C2	112.5 (2)	C14—C13—H13A	119.9
N1—C1—H1B	109.1	C12—C13—H13A	119.9
C2—C1—H1B	109.1	C13—C14—C9	121.2 (3)
N1—C1—H1A	109.1	C13—C14—H14A	119.4
C2—C1—H1A	109.1	C9—C14—H14A	119.4
H1B—C1—H1A	107.8	O2—C15—N3	125.7 (3)
C3—C2—C7	118.9 (3)	O2—C15—C16	119.6 (3)
C3—C2—C1	120.6 (2)	N3—C15—C16	114.6 (2)
C7—C2—C1	120.5 (3)	F2—C16—F2A	54.8 (4)
C4—C3—C2	120.8 (3)	F2—C16—F1	94.3 (4)
C4—C3—H3A	119.6	F2A—C16—F1	138.5 (4)
C2—C3—H3A	119.6	F2—C16—F1A	136.0 (5)
C5—C4—C3	120.1 (3)	F2A—C16—F1A	95.3 (5)
C5—C4—H4A	120.0	F1—C16—F1A	90.2 (5)
C3—C4—H4A	120.0	F2—C16—C15	113.0 (3)
C6—C5—C4	119.9 (3)	F2A—C16—C15	110.6 (3)
C6—C5—H5A	120.1	F1—C16—C15	106.8 (4)
C4—C5—H5A	120.1	F1A—C16—C15	107.3 (5)
C5—C6—C7	120.2 (3)	F2—C16—Cl1A	70.7 (4)
C5—C6—H6A	119.9	F2A—C16—Cl1A	120.0 (4)
C7—C6—H6A	119.9	F1A—C16—Cl1A	109.2 (5)
C6—C7—C2	120.1 (3)	C15—C16—Cl1A	112.5 (3)
C6—C7—H7A	119.9	F2—C16—Cl1	116.7 (4)
C2—C7—H7A	119.9	F2A—C16—Cl1	71.6 (4)
N2—C8—C9	112.7 (2)	F1—C16—Cl1	107.5 (4)
N2—C8—H8A	109.0	C15—C16—Cl1	115.7 (3)
C9—C8—H8A	109.0	Cl1A—C16—Cl1	120.6 (3)
N2—C8—H8B	109.0		
			
O1—P1—N1—C1	−45.6 (3)	C14—C9—C10—C11	0.6 (5)
N2—P1—N1—C1	−174.1 (2)	C8—C9—C10—C11	−178.7 (3)
N3—P1—N1—C1	70.4 (2)	C9—C10—C11—C12	−0.3 (5)
O1—P1—N2—C8	53.1 (2)	C10—C11—C12—C13	−0.8 (6)
N1—P1—N2—C8	−178.5 (2)	C11—C12—C13—C14	1.5 (6)
N3—P1—N2—C8	−62.8 (2)	C12—C13—C14—C9	−1.2 (6)
O1—P1—N3—C15	−175.7 (3)	C10—C9—C14—C13	0.2 (5)
N1—P1—N3—C15	60.2 (3)	C8—C9—C14—C13	179.5 (3)
N2—P1—N3—C15	−51.6 (3)	P1—N3—C15—O2	−0.6 (6)
P1—N1—C1—C2	−120.2 (2)	P1—N3—C15—C16	−179.3 (2)
N1—C1—C2—C3	−94.8 (3)	O2—C15—C16—F2	157.9 (5)
N1—C1—C2—C7	84.9 (3)	N3—C15—C16—F2	−23.3 (5)
C7—C2—C3—C4	−1.2 (4)	O2—C15—C16—F2A	−142.8 (5)
C1—C2—C3—C4	178.4 (3)	N3—C15—C16—F2A	36.0 (6)
C2—C3—C4—C5	0.4 (5)	O2—C15—C16—F1	55.6 (6)
C3—C4—C5—C6	−0.4 (5)	N3—C15—C16—F1	−125.6 (4)
C4—C5—C6—C7	1.2 (6)	O2—C15—C16—F1A	−40.0 (6)
C5—C6—C7—C2	−2.0 (6)	N3—C15—C16—F1A	138.8 (5)
C3—C2—C7—C6	2.0 (5)	O2—C15—C16—Cl1A	80.1 (5)
C1—C2—C7—C6	−177.6 (3)	N3—C15—C16—Cl1A	−101.1 (4)
P1—N2—C8—C9	−162.00 (19)	O2—C15—C16—Cl1	−64.0 (5)
N2—C8—C9—C10	5.0 (4)	N3—C15—C16—Cl1	114.8 (4)
N2—C8—C9—C14	−174.2 (3)		

### Hydrogen-bond geometry (Å, º)

**Table:**

*D*—H···*A*	*D*—H	H···*A*	*D*···*A*	*D*—H···*A*
N1—H1*N*···O1^i^	0.88 (2)	2.30 (3)	3.092 (3)	151 (3)
N2—H2*N*···O1^i^	0.86 (2)	2.05 (2)	2.867 (3)	158 (3)
N3—H3*N*···O2^ii^	0.86 (2)	2.01 (2)	2.854 (3)	166 (3)

Symmetry codes: (i) *x*, *y*−1, *z*; (ii) *x*, *y*+1, *z*.
